# Anatomic and neuropsychological findings in low-educated cognitively intact elderly from a Brazilian cohort

**DOI:** 10.1590/1980-57642018dn13-040003

**Published:** 2019

**Authors:** Maria Paula Foss, Paula Rejane Beserra Diniz, Daiane Leite da Roza, Tamar Gefen, Amanda Cook Maher, Paulo Formigheri, Carina T. Spedo, Carlos Ernesto Garrido Salmon, Vitor Tumas, José Geraldo Speciali, Antônio Carlos Santos

**Affiliations:** 1 Universidade de São Paulo, Faculdade de Medicina de Ribeirão Preto, Hospital das Clínicas de Ribeirão Preto, Neurosciences Program, Department of Neurosciences and Behavioral Sciences, Ribeirão Preto, SP, Brazil.; 2 Universidade Federal de Pernambuco (UFPE), Center of Telehealth (NUTES), Department of Clinical Medicine, Recife, PE, Brazil.; 3 University of São Paulo, Ribeirão Preto Medical School, Department of Neurosciences and Behavioral Sciences, Ribeirão Preto, SP, Brazil.; 4 Mesulam Center for Cognitive Neurology and Alzheimer’s Disease Center, Feinberg School of Medicine, Northwestern University, Chicago, IL 60611, USA.; 5 Department of Psychiatry, Neuropsychology Division, University of Michigan, Ann Arbor, MI, 48105, USA.; 6 Universidade de São Paulo, Faculdade de Medicina de Ribeirão Preto, Program of Internal Medicine, Ribeirão Preto/SP, Brazil.; 7 Universidade de São Paulo, Faculdade de Filosofia, Ciências e Letras de Ribeirão Preto (FFCLRP), Department of Physics. Ribeirão Preto, SP, Brazil.; 8 Universidade de São Paulo, Faculdade de Medicina de Ribeirão Preto, Department Internal Medicine, Radiology Division, Ribeirão Preto, SP, Brazil.

**Keywords:** cognitive reserve, educational levels, cognitive aging, MRI, neuropsychological assessment, reserva cognitiva, níveis de escolaridade, envelhecimento cognitivo, ressonância magnética, avaliação neuropsicológica

## Abstract

In elderly individuals, low educational level may represent a risk factor for the development of dementia and a proxy of cognitive reserve. Objective: This study examined the cognitive and neuroanatomic correlates of high versus low educational levels in cognitively healthy community-dwelling older adults in Brazil. Methods: Fifty-three older adults (mean age: 68±5.3 years) were divided into a “low education” group [LE; 1-4 years of education (N=33)] and “high education” group [HE; >11 years of education (N=20)]. Both groups completed a comprehensive neuropsychological battery and underwent *in vivo* structural MRI close to the time of testing. Results: Higher educational level increased the chance of having better scores on neuropsychological tests, including verbal and visual delayed recall of information, verbal learning, category fluency, global cognition, and vocabulary. Better scores on these tests were observed in the HE group relative to the LE group. Despite this, there were no group differences between MRI measures. Conclusion: Older adults with higher educational levels showed better scores on neuropsychological measures of cognition, highlighting the need for education-adjusted norms in developing countries. Given the absence of differences in structural anatomy between the groups, these findings appear to be best explained by theories of cognitive reserve.

## RESUMO

Sabe-se que baixos níveis de educação são comuns em países em desenvolvimento. Em indivíduos idosos, em particular, baixos níveis de educação podem representar um fator de risco para o desenvolvimento de demência. Objetivo: Este estudo examina os correlatos cognitivos e neuroanatômicos de escolaridade alta versus baixa, em idosos cognitivamente saudáveis,vivendo em comunidade no Brasil. Métodos: Cinquenta e três idosos (média de idade: 68±5,3) foram divididos em um grupo de “baixa escolaridade” [LE; 1-4 anos de escolaridade (N=33)] e um grupo de “alta escolaridade” [HE; >11 anos de escolaridade (N=20)]. Ambos os grupos completaram uma bateria neuropsicológica abrangente e foram submetidos à RM estrutural *in vivo* próximo à testagem. Resultados: O nível educacional aumentou a chance de se obter melhores pontuações em testes neuropsicológicos, incluindo evocação verbal e visual da informação, aprendizagem verbal, fluência de categoria, cognição global e vocabulário. Escores mais altos foram encontrados no grupo HE, em detrimento do LE. Apesar disso, não houve diferenças entre os grupos nas medidas de ressonância magnética *in vivo.* Conclusão: Idosos com maiores níveis de escolaridade apresentaram melhores pontuações nas medidas neuropsicológicas da cognição, destacando a necessidade de normas ajustadas à educação nos países em desenvolvimento. Não havendo diferenças na anatomia estrutural entre os grupos, os achados parecem ser melhor explicados pelas teorias da “reserva cognitiva”.

Low levels of education are frequent in elderly populations across the world and might represent a factor that contributes to a higher risk for developing dementia. According to current theories of cognitive reserve (CR), this may be because high levels of education are viewed as a protective factor against the development of age-related neuropathology.^1^^-^ 3 CR has emerged as a theory that represents the protective ability of optimized cognitive performance on the impact of brain pathology; that is, subjects with high reserve may show preservation of cognitive status even with some degree of neuropathology or brain insult.^4^ Proxies of CR have been defined as modifiable experiences acquired during life, the complexity of occupation attainment, active social lifestyles, and educational level - factors that are thought to provide greater reserve and modify neural networks should brain damage occur. Brain reserve (BR) theory is another possible theory that explains resistance to age-related decline; this theory posits that brains that are structurally larger and have greater neuronal density are more resistant against age-related brain changes, and that these brain features are less modifiable than socio-educational factors.^5^^,^ 6 Proxies of BR measures include intracranial volume (ICV) and brain parenchymal fraction (BPF), although the relationships between these *in vivo* measures and cognitive outcomes are not entirely clear.^7^

The present study investigates cognitive performance and *in vivo* measures of brain volume in a single Brazilian cohort comprised of cognitively intact older adults with low and high educational levels, matched by age and gender. To better understand the impact of individual differences on the clinical manifestation of age-related dementia, this study examines proxies of brain reserve (i.e., structural anatomy) versus cognitive reserve (i.e., educational level and socioeconomic status) in a singlecohort of cognitively healthy elderly adults in Brazil. 

## METHODS

### Participants

Fifty-three right-handed community-dwelling older individuals aged from 60 to 79 years (mean age, 68±5.3 years) with 1 to 15 years of education were invited to participate in the study. Subjects attended the Ribeirão Preto School of Medicine Hospital of the University of São Paulo (HCFMRPUSP), where they underwent a full clinical examination conducted by a geriatrician. All participants were deemed to be both physically and cognitively healthy, and completely independent in their activities of daily living according to scales and instruments described later in this text. The Research Ethics Committee of the HCFMRPUSP (*Processo* HC no. 9205/2004) approved the study and all subjects provided written informed consent to participate in the study. 

The sample was divided into a “lower education” group (LE group) consisting of individuals with a mean of 3.4 (±0.9) years of education (n=33) and a “higher education” group (HE group) consisting of those with a mean of 12.2 (±1.8) years of education (n=20). The individuals with 5-10 (n=11) years of education were excluded because they were a small group that did not belong either to the low or high-educated group. According to the Population Census of the Brazilian Institute of Geography and Statistics,^8^ the mean length of education for older adults (60+ years) in Brazil is 3.4 years. Thus, the LE group is more representative of the general population in Brazil. Educational level was self-reported by subjects as the total number of years of formal education received. Socioeconomic status (SES) was evaluated using the Socioeconomic Classification Instrument,^9^ a well-validated self-report questionnaire for the Brazilian population that allows reporting of the number of persons living in the same household, housing status, income, years of education, and occupation. The questionnaire yielded an SES score in the “superior,” “medium”, or “low” ranges.^9^ In general, higher scores with superior classifications represent better incomes, housing and fewer persons living in the same household, in addition to higher years of education and higher status occupations.

### Procedures

**Clinical evaluation.** Subjects were evaluated by a geriatrician to rule out medical or psychiatric conditions that could interfere with cognitive and neurological functioning. Inclusion criteria were no prior history of neurological disorders, psychiatric disorders, sensorineural deficits, chronic alcoholism (>3 drinks/day), clinical *delirium*, cardiac or pulmonary disease, endocrine-metabolic instability (e.g. diabetes mellitus), nutritional deficiencies (B12, folic acid and niacin deficiency), anemia, and use of psychotropic medication. The Brazilian version of the MINI International Neuropsychiatric Interview,^10^ Katz Activities of Daily Living scale,^11^ Clinical Dementia Rating (CDR) scale,^12^ Mini-Mental State Examination (MMSE;^13^ and laboratory tests were administered to characterize the inclusion and exclusion criteria. 

Eligible subjects were required to have median scores for their age and educational groups on the MMSE (median of 25 for individuals with 1-4 years of education; 28 for 9-11 years and 29 for >11 years of education), show no signs of psychiatric disorders,as evaluated by the MINI diagnostic interview (Brazilian version), and be independent for all activities of daily living (score of 0 on Katz activities of daily living scale, and have a CDR score of 0).

**Neuropsychological evaluation.** The neuropsychological evaluation consisted of seven tests that were appropriate for individuals with low educational levels; that is, tests that do not rely heavily on skills dependent on formal educational background, such as mathematical calculation or general knowledge. This selection also included tests sensitive to the effects of cognitive aging, such as mental processing speed,^14^ inhibitory control^15^ and memory.^16^ The cognitive battery was comprised of the Mattis Dementia Rating Scale (MDRS)^17^ to assess general cognitive impairment, the Stroop Test - Victoria version and Color Trail Test^17^^-^ 20 to evaluate attention/executive function, the Verbal Fluency (animals) to measure executive function/ language, the WMS-R Logical Memory and Visual Reproduction subtests^21^ the Rey Auditory Verbal Learning Test (RAVLT)^22^ to assess retentive memory, and the WAIS-III Vocabulary subtest^23^ as an estimate of premorbid intellectual ability.^24^ Demographic variables were collected by an informal questionnaire to assess self-reported level of education, occupation attainment, socioeconomic status (SES), and hand dominance. Current occupation was divided into three categories: retirees, homemakers, and active workers. 

The neuropsychological performance of each subject was represented by the total raw scores on the MDRS (total=144); Delayed Recall of Logical Memory from the WMS-R (WMS R, LM II; Total Raw Score) and Delayed Recall of Visual Reproduction (WMS-R, VR II; Total Raw Score); Rey Auditory Verbal Learning Test (RAVLT; Sum of learning trials), time in seconds on Stroop Test [(Part “D” (*Dot*) and Part “C” (*Color*)]; time in seconds on Color Trail Test 1 and 2 (CTT1 and CTT2); Verbal Fluency (animals; Total Raw Score) Copy of Rey Complex Figure Test (Copy RCF; Total Raw Score); and WAIS-III Vocabulary scaled score by age (mean: 10±3). 

**Image acquisition.** All subjects were scanned with 1.5T MR imaging equipment (Magnetom Vision; Siemens, Erlangen, Germany) using a commercially available quadrature head coil. The imaging protocol included a sagittal T1-weighted 3D magnetization-prepared rapid acquisition of gradient echo (MPRAGE) sequence with TR 9.7 ms, TE 4 ms, NEX 1, flip angle 12°, acquisition matrix 256 × 256, and FOV 25 cm, which produced 160 contiguous sections, each 1-mm-thick, and a 1 mm³ isotropic voxel.

### Data analysis

**Magnetic Resonance Imaging (MRI) Postprocessing.** Intracranial volume (ICV) and brain parenchymal fraction (BPF) have been previously used to study the brain reserve hypothesis.^25^ ICV estimates the head size and BPF represents the ratio of brain parenchymal to intracranial volume. Percentage of gray matter (PGM) volume is considered a valid *in vivo* measure of age-related neuropathology. Volume of white matter hypointensities (WMH) is established as a primary indicator of vascular disease in the aging brain.^26^^-^ 34 Bilateral hippocampi volume (HV) is sensitive to neurodegenerative diseases, such as Alzheimer’s dementia.^35^^-^ 37 PGM, WMH and HV were analyzed to compare reliable indicators of age-related neuropathology in low vs. high education groups. 

Volumetric measures of each brain structure were obtained using the automated FreeSurfer imaging software, version 4.5 (Martinos Center for Biomedical Imaging, Charlestown, Massachusetts, USA). This software classifies each voxel with a neuroanatomical label based on automatically estimated probabilistic information and has accuracy comparable to manual labeling (Fischl et al. 2002). This software has been recently validated in elderly subjects by Wenger et al., who showed that the quality of FreeSurfer volumetry is close to that of manual editing.^38^ All automatic segmentations were double checked by visual inspection and no mismatches were found in our data. Volumetric measures were normalized by the total ICV obtained in the FreeSurfer software and multiplied by 100 to express values as percentages.

### Statistical analysis

Student’s *t-*tests for independent samples and Fisher’s Exact tests were used to determine between-group differences across demographics. Associations between education, neuropsychological performance and MRI measures were measured using logistic regression models,^39^ where the Odds Ratio (OR) for age and gender was calculated with its respective 95% confidence intervals. All statistical analyses were performed using the statistical software SAS version 9.4.^40^ P-values ≤0.05 were considered significant.

## RESULTS

### Demographics

The LE and HE groups did not differ in age, gender or occupation (*p*>0.05). However, the two groups differed in SES level,whereby the LE group had lower SES than the HE group (*p*<0.01; Table 1). It should be noted that two individuals were not classified in the SES because the questionnaire was not applied to them. The LE group showed worse performance than the HE group on the WAIS-III Vocabulary subtest (*p*<0.01; Table 1), although all scores were within normal limits for age.

**Table 1:** Demographic characteristics.

	Groups		p-value*
	LE	HE	
**Age**	**n (mean; SD)**	**n (mean; SD)**	
	33 (69.0; 5.8)	20 (67.7; 4.3)	0.29
**Gender**	**n (%)**	**n (%)**	
Female	25 (43.1)	17 (29.3)	0.56
Male	8 (13.8)	8 (13.8)	
**SES**	**n (%)**	**n (%)**	
Medium	2 (3.8)	0 (0.0)	<0.01
Medium inferior	9 (17.4)	1 (1.9)	
Low superior	20 (38.5)	4 (7.7)	
Low inferior	1 (1.9)	13 (25.0)	
Not classified	0 (0.0)	2 (3.8)	
**Occupation**	**n (%)**	**n (%)**	
Retirees	12 (23.1)	10 (19.2)	0.67
Homemakers	11 (21.2)	5 (9.6)	
Active workers	9 (17.3)	5 (9.6)	

LE: low education group; HE: high education group; SD: standard deviation, SES: socioeconomic classification;

* p≤0.05.

### Neuropsychological performance

Association tests and logistic regression analysis demonstrated that educational level was associated with Total score of the MDRS, Visual Reproduction II WMS-R, Total score of learning trials of the RAVLT, and Animal Fluency (Table 2). The LE group showed more scores below the median for this group of cognitively healthy old adults than the HE group,which suggests that years of education was associated with performance on global cognition scores, retention of verbal and visuospatial features, learning of an auditory-verbal word list test, verbal output, and lexical definition. 

**Table 2:** Logistic regression analysis for Cognitive Reserve.

		Groups		p-value*	Crude-OR	95% CI	Adjusted-OR	95% CI
		LE n (%)	HE n (%)					
**MDRS**	<132.50	23 (41.1)	5 (8.9)	<0.01	1.0	Reference	1.0	Reference
	≥132.50	8 (14.3)	20 (35.7)		11.5	(3.2 ; 40.8)	9.7	(2.6 ; 36.2)
**LM II**	<11	18 (32.1)	7 (12.5)	0.05	1.0	Reference	1.0	Reference
	≥11	14 (25.0)	17 (30.4)		3.1	(1.0 ; 9.6)	2.4	(0.7 ; 8.5)
**VR II**	<14	21 (36.8)	7 (12.3)	0.01	1.0	Reference	1.0	Reference
	≥14	12 (21.1)	17 (29.8)		4.2	(1.3 ; 13.1)	3.5	(1.1 ; 11.4)
**RAVLT**	<39	23 (39.7)	6 (10.3)	<0.01	1.0	Reference	1.0	Reference
	≥39	10 (17.2)	19 (32.8)		7.3	(2.2 ; 23.7)	6.9	(1.9 ; 24.3)
**Vocabulary WAIS III**	<37.5	24 (42.9)	4 (7.1)	<0.01	1.0	Reference	1.0	Reference
	≥37.5	9 (16.1)	19 (33.9)		12.7	(3.3 ; 47.5)	26.5	(4.7 ; 148.9)
**Animals**	<13	21 (37.5)	3 (5.4)	<0.01	1.0	Reference	1.0	Reference
	≥13	10 (17.9)	22 (39.2)		15.4	(3.7 ; 63.8)	15.9	(3.4 ; 75.0)
**CTT1**	<78	10 (20.4)	14 (28.6)	0.08	2.9	(0.9 ; 9.5)	3.0	(0.8 ; 10.6)
	≥78	17 (34.7)	8 (16.3)		1.0	Reference	1.0	Reference
**CTT2**	<158	14 (28.6)	13 (26.5)	0.77	1.0	Reference	1.3	(0.3 ; 4.5)
	≥158	10 (20.4)	12 (24.5)		1.3	(0.4 ; 3.9)	1.0	Reference
**Stroop D**	<19.5	17 (30.4)	11 (19.6)	0.59	1.0	Reference	1.0	Reference
	≥19.5	14 (25.0)	14 (25.0)		1.5	(0.5 ; 4.4)	1.3	(0.4 ; 4.2)
**Stroop C**	<41	15 (27.3)	11 (20.0)	1.00	1.0	Reference	1.0	Reference
	≥41	16 (29.1)	13 (23.6)		1.1	(0.3 ; 3.2)	1.5	(0.4 ; 4.8)
**Copy RCF**	<31	10 (17.9)	14 (25.0)	0.10	2.7	(0.8 ; 7.9)	2.8	(0.8 ; 9.5)
	≥31	21 (37.5)	11 (19.6)		1.0	Reference	1.0	Reference

* p-value for Fisher's Exact Test; 95% CI=95% confidence interval. MDRS: Mattis Dementia Rating Scale; LM II: Logical Memory II; VR II: Visual Reproduction II; RAVLT: Rey Auditory Verbal Learning Test; WAIS III: Wechsler Adult Intelligence Scale III; CTT 1 & 2: Color Trail Test 1 & 2; Copy RCF: Copy Rey Complex Figure.

An adjusted logistic regression model (Table 2) showed that high education increased the chance of scoring above the median on the Vocabulary WAIS III by 26.5, even when adjusted for age and gender (95% CI: 4.7; 148.9). Similarly, education increased the chance of scoring above the median in verbal fluency animals (95% CI: 3.4; 75) by 15.6, and the chance of higher MDRS total score (95% CI: 2.6; 36.2) by 9.7, the chance of better total RAVLT score (95% CI: 1.9; 24.3) by 6.9,chances on delayed recall of Visual Reproduction (95% CI: 1.1; 11.4) by 3.5 and the chance on delayed recall of Logical Memory (95% CI: 0.7; 8.5) by 2.4, controlling Odds Ratio for age and gender. Years of education was not, however, associated with performance on the CTT 1 & 2, Stroop D & C, or the Copy of RCF tests. This suggests that performance on measures of concentration, control inhibition, divided attention, and complex figure copy might not be influenced by educational level.

### Brain reserve

Subsequent analyses examining associations between educational groups and MRI measures was performed to determine whether *in vivo* MRI measures, including percentage of GM (%GM), ICV, BPF, and bilateral hippocampus volumes were associated with educational level. There were no statistically significant associations between educational level and any structural MRI measures (*p*>0.05; see Table 3). 

**Table 3:** Frequency tables and association test for proxies of brain reserve.

		Groups		p-value*
		LE n (%)	HE n (%)	
**%GM**	<0.22	10 (25.6)	12 (30.8)	1.00
	≥0.22	7 (18.0)	10 (25.6)	
**ICV**	<1291.6	12 (30.8)	10 (25.6)	0.52
	≥1291.6	7 (18.0)	10 (25.6)	
**BPF**	<74.6	11 (27.5)	12 (30.0)	1.0
	≥74.6	9 (22.5)	8 (20.0)	
**Left hippocampus**	<0.23	13 (32.5)	10 (25.0)	0.52
	≥0.23	7 (17.5)	10 (25.0)	
**Right hippocampus**	<0.24	11 (27.5)	12 (30.0)	1.00
	≥0.24	9 (22.5)	8 (20.0)	
**WMH**	<0.12	10 (25.0)	13 (32.5)	0.52
	≥0.12	10 (25.0)	7 (17.5)	

* p-value for Fisher's Exact Test. % GM: Percentage of Gray Matter; ICV: Intracranial Volume; BPF: Brain Parenchymal Fraction; WMH: White Matter Hypointensities.

## DISCUSSION

Low educational level, socioeconomic status, and poor income are common among elderly populations in developing countries. Educational level is often used as a proxy measure for CR, which may explain why some cognitively healthy elderly individuals with high educational levels do not exhibit symptoms of cognitive decline, despite showing significant postmortem neuropathology.^41^ Studies that investigate reserve theories typically rely on recruitment from highly educated groups of participants from developed countries, which do not adequately represent the global elderly population. In this study, we investigated differences between older adults with a “lower education” (LE) level (1-4 formal years), consistent with the average educational level of older Brazilian adults, and those with “higher education” (HE) (11+ formal years), more consistent with average education in developed countries, but atypical for older Brazilian adults. We investigated the effects of CR, operationalized as years of formal education, and BR, operationalized as intracranial volume (ICV) and brain parenchymal fraction (BPF), on cognitive performance within this cohort of older Brazilian adults. The goal was to determine whether educational levels were linked to factors implicated in cognitive versus brain reserve theories.

First, as expected, the LE group showed significantly lower socioeconomic status compared to the HE group. Occupation and premorbid intellectual estimates are also important variables that are strongly associated with the concept of reserve.^42^^,^ 43 The groups did not differ in their current occupation and most subjects from both groups were retired, although the LE group contained a significantly greater number of homemakers.

Significant associations were found between educational level and cognitive performance, including measures of global cognition, memory, and language (i.e., verbal fluency animals and WAIS III Vocabulary). These findings are consistent with other studies conducted in developing countries in individuals with low educational levels,^44^^,^ 45 where they have mistakenly been regarded as cognitively impaired due to false-positive interpretation of neuropsychological test results. In a similar study, Huang and Zhou (2013) showed that completing primary school significantly increased cognitive test scores, especially in episodic memory, as measured by delayed verbal and visuospatial recall tasks. Interestingly, the association between educational level and cognitive test performance was not universal across all tests in the present study, as no associations were evident between educational level and measures of concentration, control inhibition, divided attention and copy of a complex figure. In another study, two groups of older adults with different educational levels (1-7 years and >7 years) were compared, showing differences in attention measures, in contrast with our findings.^46^ Differences in the classification of high versus low levels of education may account for contrasting findings among studies. 

In the present study, between-group neuropsychological differences were not associated with differences in structural brain measures, including BPF, PGM, hippocampal volume, and WMH, all of which have been implicated in prior studies of cognitive decline.^26^^-^ 37 ^,^ 47 ^,^ 48 Similarly, neuropsychological differences could not be explained by differences in ICV which, like BPF, is a popular measure used in studies investigating brain reserve.^49^ Thus, our results conflict with other studies that show differences in global or regional brain measures related to educational level.^50^^-^ 52 This disparity could be attributed to the fact that a number of these studies used a region of interest approach to structural imaging, and that educational levels were in fact higher than the thresholds used in the current study. 

One limitation of our study is that the selection criteria excluded individuals with common medical conditions such as diabetes, perhaps impacting the generalizability of findings. This is especially relevant, given that community-dwelling individuals with low socio-economic status are typically have fewer health care options and a higher prevalence of age-related ailments.^53^^-^ 55 It is also possible that study participants who appeared cognitively healthy had underlying brain neuropathology that may contribute to worse cognitive performance, despite the absence of clinical dementia symptoms. Given that postmortem data was unavailable, we cannot confirm this possibility. However, a prior investigation from the Brazilian Aging Study showed an association between level of education and cognitive impairment, even when controlling for postmortem neuropathology, demographics and socioeconomic variables,^1^ suggesting that education contributes to CR. 

The findings of the current study suggest that low educational level, coupled with poor socioeconomic level, is correlated with lower neuropsychological performance in older age. This difference in neuropsychological performance was not accounted for by differences in structural brain integrity, suggesting that the brain reserve theory may not explain the preservation of cognitive function among healthy older Brazilian adults. Indeed, our findings are more aligned with theories of CR, in that educational level appeared to be a more robust factor contributing to differences in neuropsychological performance between the low and high education groups. We conclude that lower educational level does not necessarily result in changes to brain structure in old age, but does impact cognition. The findings of this study have important implications for the accurate diagnosis of elderly individuals with low levels of education-a common demographic in developing countries such as Brazil. In general, this can contribute to our understanding about how educational level affects brain morphology and cognition differently, furthering the debate between inherent predispositions versus the influences of life experiences and environment. Our results have yet to be confirmed in brain-damaged individuals, such as those with Alzheimer’s disease or other neurodegenerative conditions.

Our sample included a single cohort of carefully selected community-dwelling elderly individuals from Brazil. Future studies will benefit from the inclusion of similarly selected individuals from other developing countries worldwide. Studies of this kind can help to further elucidate the critical link between educational levels, cognitive functioning, and age-related neurodegeneration, with the ultimate goal of improving accurate diagnosis and prognoses in geriatric populations globally. 
